# Pleiotropic Effects of Epithelial Mesenchymal Crosstalk on Head and Neck Cancer: EMT and beyond

**DOI:** 10.1007/s12307-019-00228-y

**Published:** 2019-07-11

**Authors:** T. B. Steinbichler, D. Savic, D. Dejaco, A. Romani, B. Kofler, I. I. Skvortsova, H. Riechelmann, J. Dudas

**Affiliations:** 1 Department of Otorhinolaryngology, Head and Neck Surgery, Medical University of Innsbruck, Anichstr.35, A-6020 Innsbruck, Austria; 2 Department of Radiation Oncology, Medical University of Innsbruck, Anichstr.35, A-6020 Innsbruck, Austria

**Keywords:** IL-6, EMT, JAK/STAT3/Snail signalling, radiochemotherapy, therapy resistance, conditioned medium

## Abstract

Epithelial mesenchymal crosstalk (EMC) describes the interaction of the tumor stroma and associated fibroblasts with epithelial cancer cells. In this study we analysed the effects of EMC on head and neck cancer cells. In tumor cell lines EMC was induced using media conditioned from a mix-culture of cancer cells and fibroblasts. Cell proliferation and chemotherapy response were assessed using direct cell counting. Flow cytometry, immunohistochemistry of markers of epithelial-mesenchymal transition (EMT) and subsequent TissueFaxs™ acquisition and quantification and western blot analysis were performed. Holotomographic microscopy imaging was used to visualize the effects of EMC on Cisplatin response of SCC-25 cells. EMC induced a hybrid epithelial-mesenchymal phenotype in SCC-25 cells with co-expression of vimentin and cytokeratin. This hybrid phenotype was associated with chemotherapy resistance and increased proliferation of the cells. The EMC conditioned medium led to an activation of the IL-6/STAT3 pathway with subsequent phosphorylation of STAT3. EMC induced a hybrid epithelial-mesenchymal phenotype in HNSCC cells accompanied by increased therapy resistance and cell proliferation. The IL-6/STAT3 pathway might be one of the major pathways involved in these EMC-related effects.

## Introduction

Tumor cells constantly interact with their surrounding tumor microenvironment. Apart from tumor cells the tumor microenvironment consists of blood vessels, extracellular matrix, other non-malignant cells like fibroblasts, immune cells, pericytes or adipocytes and signalling molecules. Especial fibroblasts secrete growth factors and chemokines that support growth and survival of tumor cells [1].

This interaction of the tumor stroma and associated fibroblasts with tumor cells is also referred to as epithelial-mesenchymal crosstalk (EMC). EMC relies on paracrine signaling, cell-cell interactions and cell-matrix interactions. EMC related effects include increased cancer cell mobility [2], invasiveness [2, 3], acquisition of a mesenchymal phenotype (EMT) [4], generation of cells with stem-like properties and radiochemotherapy resistance [4, 5]. These effects might be associated with epithelial to mesenchymal transition (EMT), but also occur without EMT meaning that EMT is just one of the effects caused by EMC [5]. In EMT, epithelial cells acquire mesenchymal characteristics under the influence of the surrounding microenvironment. EMT is closely linked to embryonic development, wound healing, tissue fibrosis in chronic inflammation and cancer progression [6]. During EMT, epithelial cells lose their junctions and polarity, re-organize their cytoskeleton, undergo a change in the signalling programmes that define cell shape and re-programme gene expression; this increases the motility of individual cells and enables the development of an invasive phenotype [7–9]. Molecular markers of EMT are decreased expression of epithelial cadherin (E-cadherin) and cytokeratines and an increased expression of vimentin and neural cadherin (N-cadherin) [10, 11]. EMT is further associated with the induction of cancer stem cells (CSC) which efficiently are able to generate new tumors or form metastasis. Through EMT differentiated cancer cells can reversibly transit into CSC and vice versa enabling adaption to the varying requirements of tumor progression and the metastatic cascade [12, 13].

One of the major signalling factors in EMC is interleukin-6 (IL-6) [14–16]. IL-6 is a pleiotropic cytokine, widely appreciated as a major regulator of the acute phase response, yet harbouring numerous functions outside the immune system, including lipid metabolism, insulin resistance, mitochondrial function, and neuroendocrine regulation [16, 17]. IL-6 signaling is mediated by a heterodimeric receptor complex comprised of the ligand-binding subunit and the signal-transducing subunit [14]. IL-6 is one of the main chemokines present in serum samples of head and neck cancer patients and elevated IL-6 levels independently predict tumor recurrence, poor survival and tumor metastasis [18]. The tumor-promoting activities of IL-6 are manifold and include the evasion of growth suppression by regulating the TP53 gene [19], mediating resistance against cell death [20, 21], increasing stemness of tumor cells [22, 23], and mediating tumor invasion and metastasis through EMT via the JAK/STAT3/Snail signaling pathway [24, 25].

In this study we analysed the effect of EMC, especially mediated through IL-6 in head and neck cancer cells.

## Methods

### Patient Samples, Immunohistochemistry

The procedures followed were in accordance with the ethical standards of the committee on human experimentation of the institution and in accord with the Helsinki Declaration of 1975 as revised in 1983. Permission was obtained from the local ethics committee to collect pretreatment biopsy samples for molecular biological investigation, paraffin embedding, sectioning, and immunohistochemical analysis (Reference Number: UN4428 303/4.14, 26 July 2011). Written informed consent was obtained from all patients. Immunohistochemical analysis of pan-cytokeratin, vimentin and α-SMA was performed in randomly selected specimens of incident locally advanced HNSCC patients treated between March 2010 and October 2017 at the Department of Otorhinolaryngology—Head and Neck Surgery, Medical University of Innsbruck. Pretreatment tumor samples were obtained during diagnostic panendoscopy. Patient samples were paraffin embedded, sectioned, and immuno-stained as published before [26].

### Immunohistochemistry

Indirect immunofluorescence staining was performed using pre-diluted mouse monoclonal pan-cytokeratin antibody (cat. nr. 760–2595, Roche Ventana, Mannheim, Germany) and 1:100 diluted rabbit polyclonal anti-vimentin, clone SP20 antibody (Linaris, Dossenheim, Germany), 1:200 diluted mouse monoclonal α-SMA antibody (Cat. Nr. A5228, Sigma, Darmstadt, Germany). Secondary anti-mouse and anti-rabbit Alexa Fluor™ 488 or 594 conjugated antibodies for detection of the immunoreaction were purchased from Molecular Probes (Life Technologies, Darmstadt, Germany). All antibodies were diluted as suggested by the manufacturers. The immunofluorescent-stained slides were cell nuclei counterstained by DAPI (Molecular Probes) and covered in Vectashield Vibrance (Vector Laboratories, Burlingame, CA, USA). Immunohistochemical reactions were acquired in TissueFaxs system and quantified using TissueQuest software as published before [26].

### Cell Lines

Human gingival fibroblasts (HGF) and Detroit 562 cells were purchased from Cell Line Service, Eppelheim, Germany [10, 27]. SCC-25 cells were purchased from German Collection of Microorganisms (DSMZ, Braunschweig, Germany). All cells were cultured in DMEM/F12 (PAA, Pasching, Austria) supplemented with 10% FBS (PAA), 2 mM l-glutamine, 100 units/ml penicillin, and 100 μg/ml streptomycin [10].

### Production of Conditioned Medium

For the production of EMC-conditioned medium (EMC-CM), 4 × 10^4^ SCC-25 or Detroit 562 cells/ml and 1 × 10^4^ HGF cells/ml were plated in 250 ml cell culture flasks and cultured for 72 h in 15 ml foetal bovine serum-containing medium (1:1 mix of DMEM/F12 (PAA) and DMEM-low glucose (PAA) supplemented with 10% foetal bovine serum (FBS) (PAA), 2 mM l-glutamine, 100 units/ml penicillin, and 100 μg/ml streptomycin) [4, 5]. Then the cells were washed twice with Dulbecco’s Phosphate-Buffered Saline (DPBS) (Biowhittaker®, Oud-Heverlee, Belgium) and the serum-containing medium was replaced by 15 ml albumin-containing medium (7,5 ml DMEM/F12 (PAA) and 7,5 ml DMEM-low glucose (PAA) supplemented with bovine serum albumin (BSA, PAA) (0.4 g albumin/100 ml medium) replacing the protein content of 10% FBS, 2 mM l-glutamine, 100 units/ml penicillin, and 100 μg/ml streptomycin). Albumin-containing medium was left 48 h on the mix-culture allowing interacting epithelial cells and fibroblasts to secrete EMC-related factors into the medium [4, 5]. Afterwards, EMC-CM was collected and cells were counted. EMC-CM was portioned according to cell numbers as described by Hassona and colleagues [28]. EMC-CM cultured cells were used for flow cytometry, or embedded in agarose and paraffin and subsequently used for indirect fluorescent immunohistochemistry as described above. Medium conditioned from SCC-25, Detroit 562 cells or HGF fibroblasts was produced in a similar way but 5 × 10^4^ /ml cells were plated in 250 ml cell culture flasks and mono-cultured in serum containing medium for three days and afterwards in albumin-containing medium as described above [2]. The CM was sterile-filtered and stored at −80 °C.

### Flow Cytometry of Mixed Cultured Cells

EMC culture was mixed as described above (4 × 10^4^ SCC-25 cells/ml and 1 × 10^4^ HGF cells/ml) and processed immediately for flow cytometry or was cultured for 5 days (72 h with serum, 48 h serum-free). EMC-CM was collected, filtered and the cultured cells were processed for flow cytometry using PerFix-nc kit of Beckman Coulter (Marseille, France). Cells were fixed in formaldehyde containing solution of the kit, permeabilysed by the kit solution “2” and stained with conjugated antibodies as follows: 7 μl cytokeratin 18-Alexa Fluor 488 / 10^7^ cells (Invitrogen, Darmstadt, Germany), vimentin Phycoerythrin (PE) 1: 10 dilution (Exbio, Prague, Czech Republic), SMA–AF 488 1:300 dilution (eBioscience, Darmstadt, Germany). Isotype matching immunoglobulins with the same conjugates were purchased from Exbio, eBioscience and Invitrogen. PE and AF antibodies were used combined in the flow cytometry. For preparation of the cell suspension samples the instructions of PerFix nc kit kit were followed. The fixed and immunostained cells were investigated in CytoFLEX flow cytometer of Beckman Coulter (Brea, CA, USA) following the instructions of the manufacturer.

### Stimulation of SCC-25/ Detroit 562 Cells with CM and IL-6

SCC-25/Detroit 562 cells were treated with 7 ml EMC-CM per 50 ml cell culture flask for 72 h. The medium was changed daily. To assess the effects of IL-6 SCC-25/ Detroit 562 cells were cultivated in albumin-containing medium supplemented with IL-6 at 50 ng/ml (RnD Systems, Biomedica, Vienna, Austria). The concentration of IL-6 was based on pre-experiments and on a publication of Sullivan and colleagues [29]. Exposure conditions were the same as in the first experimental group, i.e., IL-6 supplemented medium was used over a period of 72 h. At the end of the stimulation period, cells were used for immunohistochemistry, protein isolation and MTT assays respectively.

### Protein Isolation and Western Blotting

Following treatments SCC-25 and Detroit 562 cells were washed twice with cold DPBS and scraped in 500 μl RIPA-buffer (50 mM Tris HCl/pH:7.4, 1 mM EDTA, 0.5 mM EGTA, 1% Triton X-100, 0.25% sodium deoxycholate, 0.1% sodium dodecylsulfate, 150 mM NaCl, 10 mM NaF, 1 mM PMSF; 10 μl proteinase inhibitors of Invitrogen “Halt Inhibitors mix/ml RIPA buffer)/culture dish. The cell suspension was vortexed and incubated three-times for 15 min on ice, homogenized in 22G needles and centrifuged at 15000 g, 15 min, 4 °C. The cleared supernatant was subjected to protein concentration measurement using the Pierce 660 nm protein assay (Pierce, Rochford, IL, USA) according to the instructions of the manufacturer. 10 μg protein from all samples was subsequently processed for western blot, using Invitrogen NuPage gels, electrophoresis and blotting system. Western blot detection was done as published before [10], using primary antibodies: rabbit monoclonal anti-STAT (1:1000) or phospho–STAT (1:2000) and positive controls from Cell Signaling Technologies (Danvers, MA, USA), mouse monoclonal anti-Slug at 125 ng/ml (Cat. Nr. 564614, BD Pharmingen, Szabo Scandic, Vienna Austria), rabbit polyclonal anti-Snail1:1000 (Cat. Nr. PA5–11923, Pierce, Darmstadt, Germany), mouse monoclonal anti-GAPDH, 1:100(Santa Cruz Biotechnology, Szabo Scandic, Vienna Austria), anti mouse monoclonal β-actin 1: 2 × 10^4^ (Proteintech, Manchester, UK). For signal detection horseradish peroxidase coupled matched secondary antibodies (1:1000 and 1:2000 dilutions, depending on the instruction of the manufacturer) and chemiluminescent substrate of Thermo Fisher Scientific (Darmstadt, Germany,) were used in conditions suggested by the manufacturer. The chemilumescence signal was imaged by an Azure C500 documentation system (Biomedica, Vienna, Austria).

### Paraffin Embedding of Cultured Cells

Routinely cultured cell lines and mixed cultures were collected by centrifugation and embedded as cell pellets in agarose as published before [26, 30] and modified as follows: Cells were harvested by centrifugation at 290 × *g* for 10 min at 4 °C, and the resulting pellet was fixed in 10 mL neutral buffered 4% formaldehyde solution (SAV Liquid Production Ltd., Flintsbach am Inn, Germany). After fixation the cells were centrifuged at 400 × *g* for 10 min at room temperature. The cell pellet was resuspended in 300 μL PBS, transferred to Eppendorf tube (1.5 mL), and kept on ice. Low melting point agarose (gelling temperature point 34–37 °C) was prepared in PBS as 3% solution in labor glassware by microwave warming and it was equilibrated in a thermoblock to 65 °C for at least 30 min. The 300 μL PBS-cell suspension was also equilibrated to 65 °C for not more than 10 min. Then, 600 μL melted equilibrated agarose was pipetted to the cell suspension, followed by spinning at 2000 ×* g* for 5 min at room temperature. After that, the tube was placed on ice, the cell pellet was trimmed, and it was placed in embedding cassette. The cell pellet in the cassette was stored in PBS containing 0.05–0.1% sodium azide until embedded in paraffin as published in detail before [26].

Similar to the tissue sections, from the cell pellets 5 μm thick sections were cut. The cell sections did not contain any overlaps as the cells were distributed. The cell sections were stained immunohistochemically identical to the tissue sections. The percentage of positive cells for the required reaction was identified after scanning the sections in the TissueFaxs system and evaluating with Tissuequest software [26].

### Holotomographic Microscopy

10^5^ SCC-25 cells/ml were plated in cell culture dishes (1.5ml/dish) (IbiDi Ltd., Planegg, Germany) in DMEM/12 supplemented with 10% FBS for 24 h. Afterwards the cells were washed with PBS and cultured in albumine medium or EMC-CM containing IC_50_ (6.2 μM) Cisplatin (Ebewe, Unterach am Attersee, Austria, Ref. 4) for 3 days. Morphological properties of albumin-medium/cisplatin and EMC-CM/cisplatin cultured cells were assessed by live cell imaging using the Nanolive 3D Cell Explorer holotomographic microscope (Ecublens, Switzerland) without any additional materials or components.

## Results

### EMC and its Cells

**Fig. 1:**
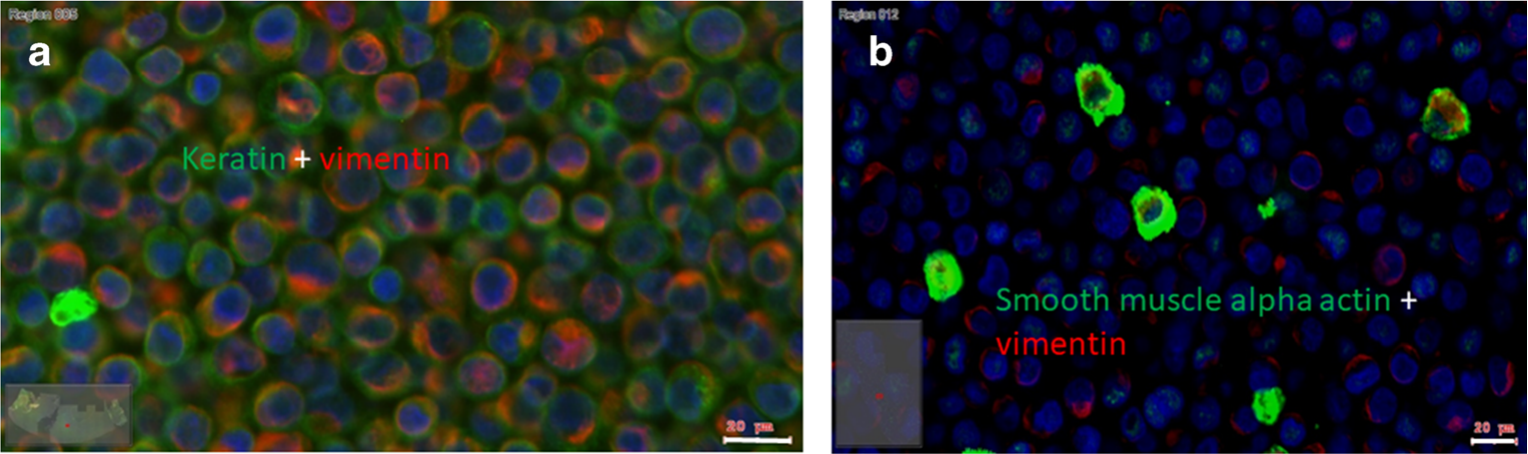
**EMC model of HNSCC in cell culture.** The mixed culture of SCC-25 cells and HGF fibroblasts functioned as model for EMC. After cell culture and production of conditioned medium (EMC-CM) the cells were embedded in agarose and in paraffin, sectioned and immunostained using anti-pan-cytokeratin (green) and vimentin (red) antibodies (**a**) or smooth muscle alpha actin (SMA, green) and vimentin (red) antibodies (**b**). The most abundant component of EMC in cell culture is the EMT cell, showing positive reaction for both pan-cytokeratin and vimentin (coloured in yellow or orange), but SMA^+^ myofibroblasts (**B**, green) are also detected in this complex. Bars: 20 μm (*n* = 5)

**Fig. 2:**
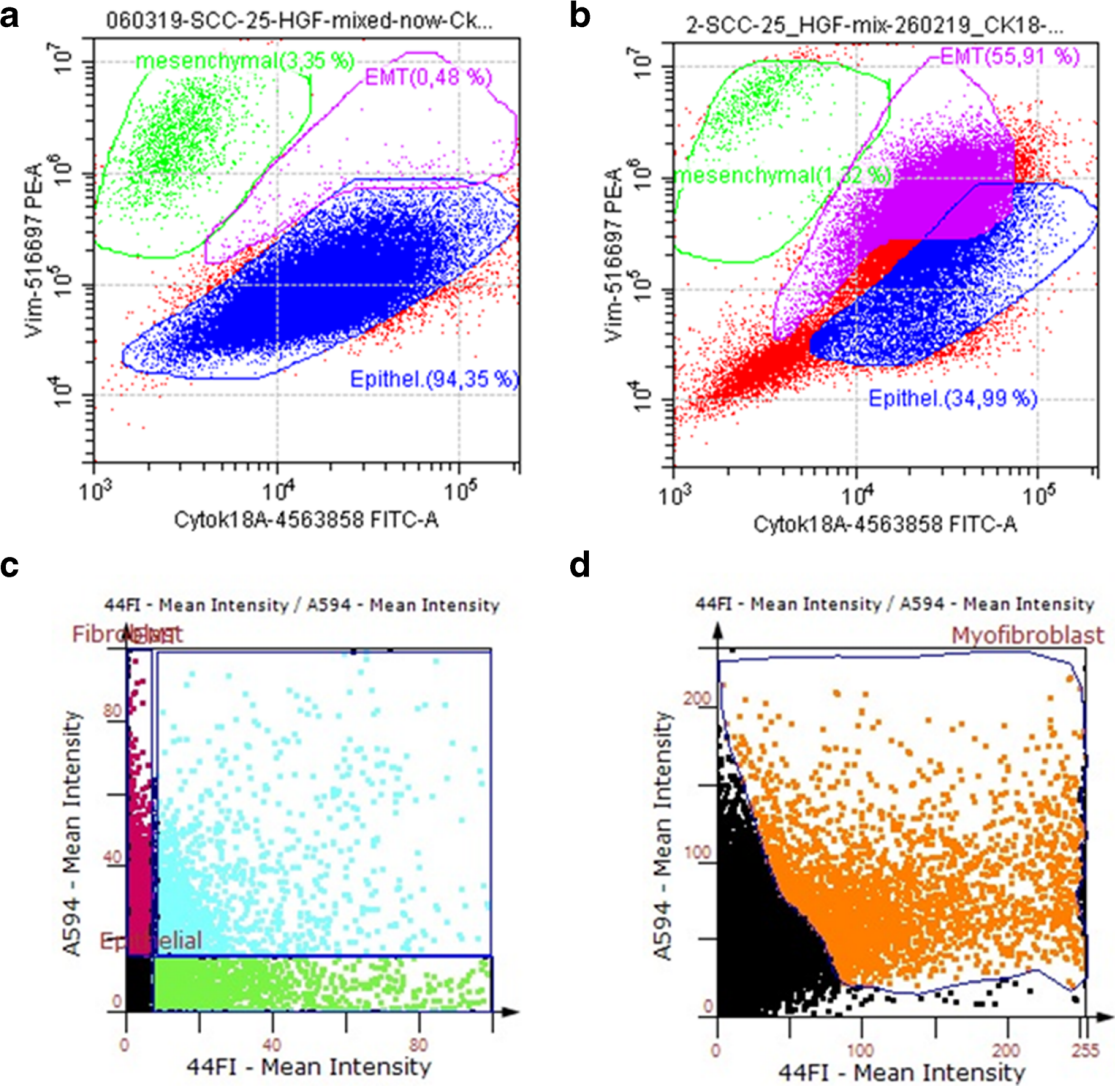
**Flow cytometry and TissueFaxs™/ TissueQuest™ analysis of the EMC model of HNSCC in cell culture. A**) SCC-25 oral and HGF fibroblasts were cultured separately and were mixed before flow cytometry. Cells were fixed and stained using the PerFix-nc kit of Beckman Coulter and cytokeratin-18-Alexa Fluor 488, and vimentin- Phycoerythrin direct conjugated antibodies. This sample was used to set the epithelial (blue) and fibroblast (green) gates in the CytoFLEX™ flow cytometer (**A**). **B**) If SCC-25 cells and HGF fibroblasts were cultured for production of EMC-CM, the most abundant component of this mixed EMC-culture was the cytokeratin-18–vimentin double positive cell type, which represents the EMT cell (labelled as magenta in panel **B)**. **C-D**) Agarose and paraffin embedding and subsequent TissueFaxs™ aquisition and TissueQuest™ evaluation of the mix cultured SCC-25 and HGF cells of EMC showed comparable results to **B**, providing an abundant pan-cytokeratin (detected with Alexa Fluor 488; x-axis on **C** photographed in 44Fl channel)–vimentin (detected with Alexa Fluor 594; y-axis on **C** photographed in A594 channel) double positive cell population (light blue on panel **C**). If the embedded cells were stained with vimentin (detected with Alexa Fluor 594; y-axis on **D** photographed in A594 channel) and SMA (detected with Alexa Fluor 488; x-axis on **D** photographed in 44Fl channel); besides the double–negative epithelial cell population and only vimentin–positive fibroblasts (black gate on panel **D**) a significant vimentin–SMA–double positive cell population (orange gate on panel **D**) was detectable, which is identified as cancer-associated fibroblast (n = 5)

Mixed culture of SCC-25 and HGF cells, functioned as model for EMC (Fig. 1 and 2). During direct mix culture of SCC-25 cells and HGF fibroblasts for production of EMC-CM, the main component was a high cytokeratin and high vimentin expressing cell population (Fig. 1 and 2), which is considered as mesenchymal trans-differentiated epithelial cell type (EMC-cell) [6].

A further identified cell population showed high vimentin and SMA levels and might represent cancer-associated fibroblasts (CAFs) (Fig. 1 and 2). SMA is a marker widely used for the identification of CAFs, even if it is also expressed in vascular smooth muscle cells, pericytes, and myoepithelial cells [31].

To study this phenomena more closely SCC-25 cells and HGF fibroblasts were cultured separately and were mixed just before flow cytometry. This sample was used to set separate gates for epithelial and the mesenchymal cells in the CytoFLEX™ flow cytometer. Most cells settled in the epithelial gate, meaning they expressed cytokeratin-18 (94.35%) and in the mesenchymal gate (3.35%), meaning they expressed vimentin. Cells with an EMC phenotype expressing both cytokeratin-18 and vimentin were rare (0.48%) (Fig. 2).

After five days of mix-cultivation of SCC-25 cells and HGF cells the distribution of these three cell phenotypes changed completely. The most abundant cell type was the EMC-cytokeratin-18 and vimentin double positive cell (55.91%). 34.99% of the cells expressed only cytokeratin-18 and 1.32% expressed only vimentin (Fig. 2).

**Fig. 3:**
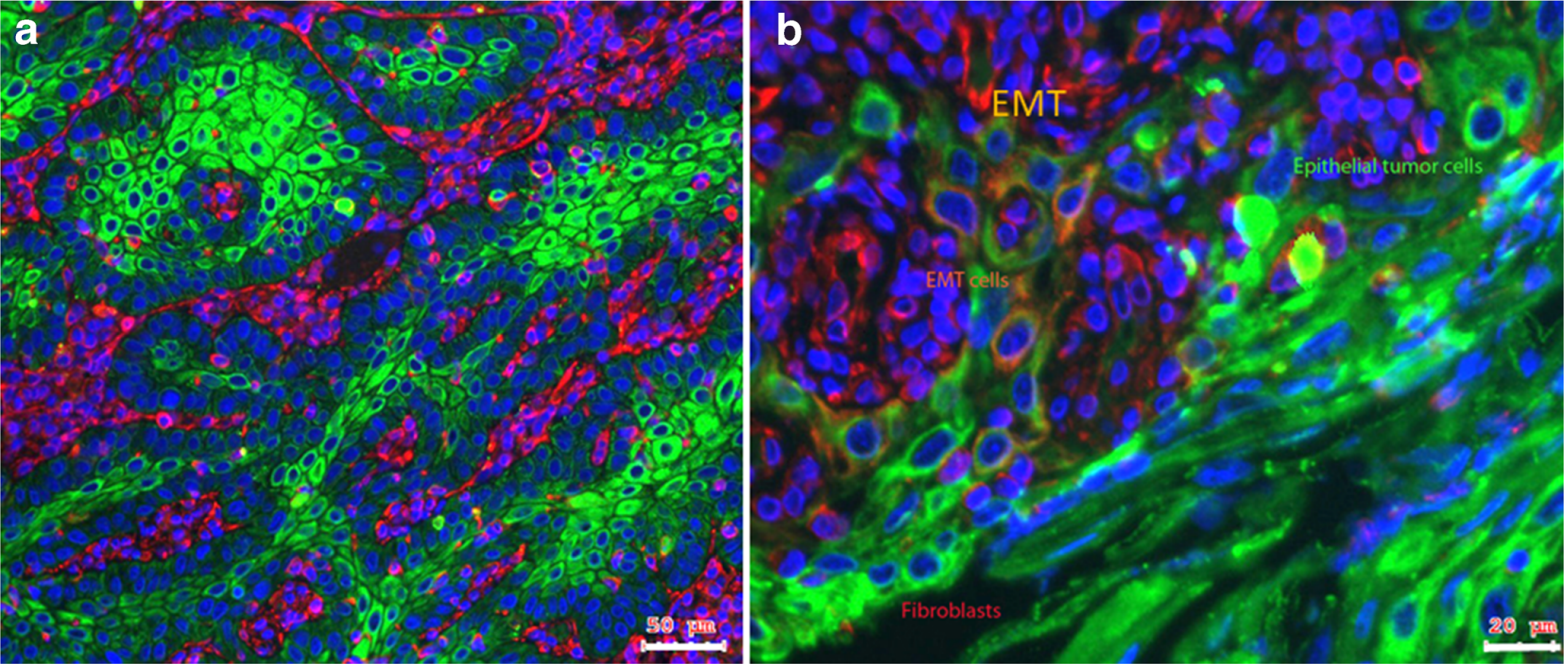
**EMC in HNSCC tissue of oral cavity. a)** Immunohistochemical reaction of pan-cytokeratin (green) and vimentin (red) in HNSCC tissue. The tumour cell nests (green) are surrounded by fibroblasts (red) and seemed to build functional units in head and neck cancer. **b)** In several HNSCC samples, this structure was replaced by a mixture of epithelial and mesenchymal cells (**B**) as pan – cytokeratin – positive epithelial tumour cells (green) and vimentin-positive fibroblasts (red), but also yellow–orange double positive cells, which are mesenchymal transdifferentiated epithelial cells (EMT). Bar represents 50 μm in panel A and 20 μm in panel B (n = 5)

Furthermore if the embedded cells were stained with vimentin and SMA-besides the double-negative epithelial cells and the only vimentin-positive fibroblasts-a significant vimentin/SMA double positive cell population was observed, which might represent CAFs (Fig. 2). To further demonstrate that this double-positive ‘hybrid’ epithelial-mesenchymal cell population is not just a laboratory phenomena but could be also found in vivo in head and neck cancer patients, tissue biopsies were analysed for these ‘hybrid’ epithelial-mesenchymal cells. Immunohistochemistry revealed tumor cell nests stained with pan-cytokeratin, that are surrounded by fibroblasts stained with vimentin and build functional units in head and neck cancer. Nevertheless, in several HNSCC samples, this structure was replaced by a mixture of epithelial and mesenchymal cells containing a significant amount of double positive ‘hybrid’ EMC cells (Fig. 3).

### EMC Increased Cell Proliferation and Cisplatin Resistance

SCC-25 and Detroit 562 cells were treated with EMC-CM or with media conditioned from a mono-culture of SCC-25/ Detroit 562 cells.

**Fig. 4:**
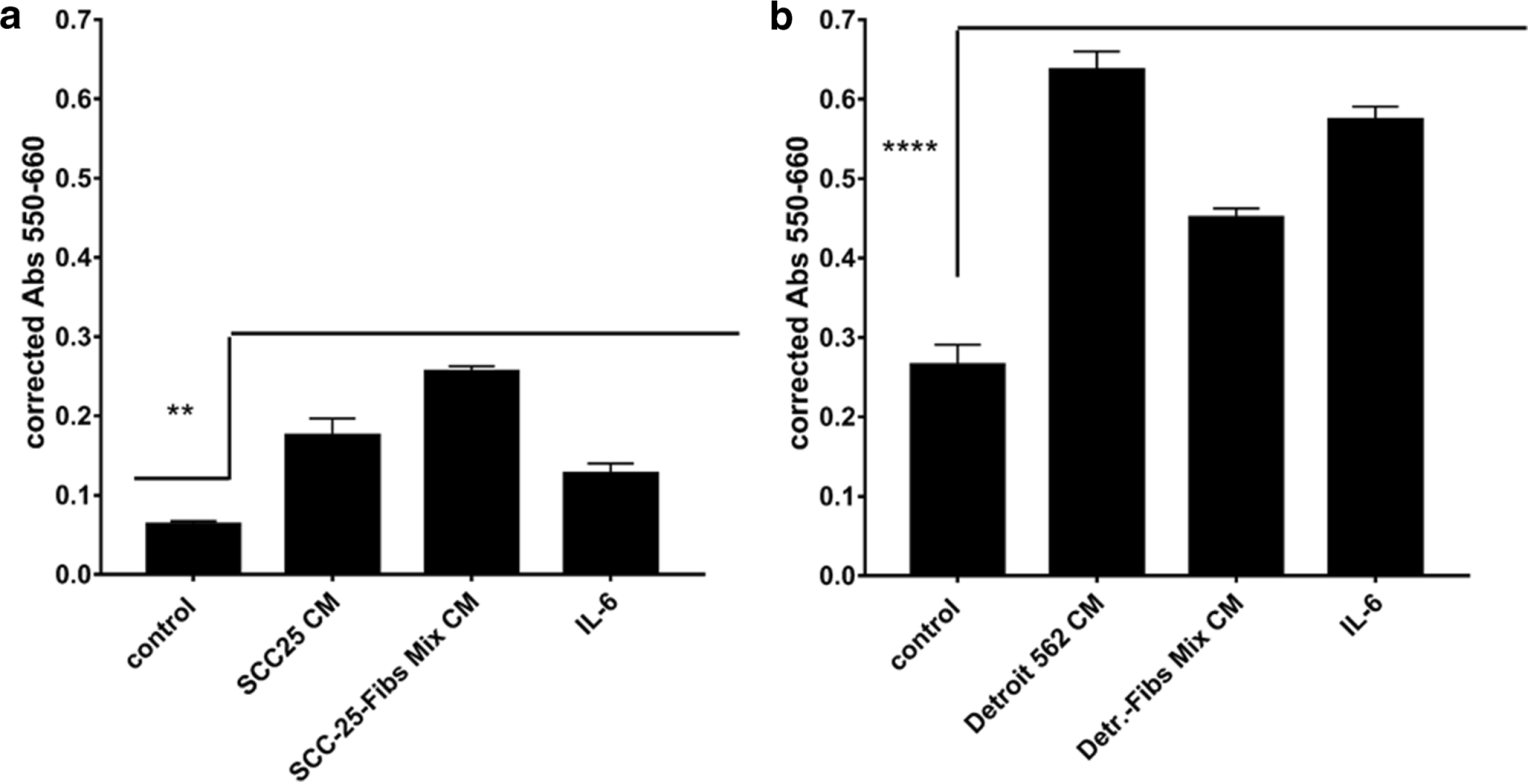
**EMC increased cell growth in HNSCC cells.** SCC-25 (**a**) and Detroit 562 (**b**) cells were treated with conditioned medium of single tumor cells, single fibroblasts and mix culture of tumor cells and fibroblasts (EMC). All conditioned media provided a significantly increased cell proliferation compared to the controls measured by cell couting, but in SCC-25 cells the highest effect was observed after treatment with EMC-conditioned medium (EMC-CM; SCC-25-Fibs Mix CM). Using cytokine/chemokine array the most abundant cytokine especially in EMC-CM was interleukin-6 (IL-6), which also supported increased cell growth in both SCC-25 and Detroit 562 cells if used for treatment of the cells at 50 ng/ml. In both panels A and B, the used statistical test was One Way Analysis of Variance (ANOVA) with multiple comparisons of all treatments to controls using Graphpad Prism 7.00 (*n* = 24)

All of these treatment arms significantly increased cell growth measured by MTT test (Fig. 4).

A cytokine array from EMC-CM was performed and the most abundant cytokine in EMC-CM was IL-6. Treatment with albumin media containing IL-6 at a concentration of 50 ng/ml significantly increased cell growth, too, but in SCC-25 cells, to a lower extent than EMC-CM (Fig. 4). Further cytokines highly expressed in EMC-CM were brain-derived neurotrophic factor (BNDF), interleukin-2 (IL-2), B lymphocyte chemoattractant (BLC) and Eotaxin 2.

In contrast, in indirect co-culture conditioned media fibroblast growth factor (FGF)-6 and −7, interleukin 16, stromal derived factor-1 (SDF-1) and tumor necrosis factor-α (TNF-α) were highly expressed. In SCC-25 conditioned media angiopoietin (ANG), CC-chemokine ligand 23 (CCL23), BLC and Eotaxin 1 were highly expressed and in HGF-fibroblast conditioned media stem cell factor (SCF) was the most abundant cytokine.

**Fig. 5:**
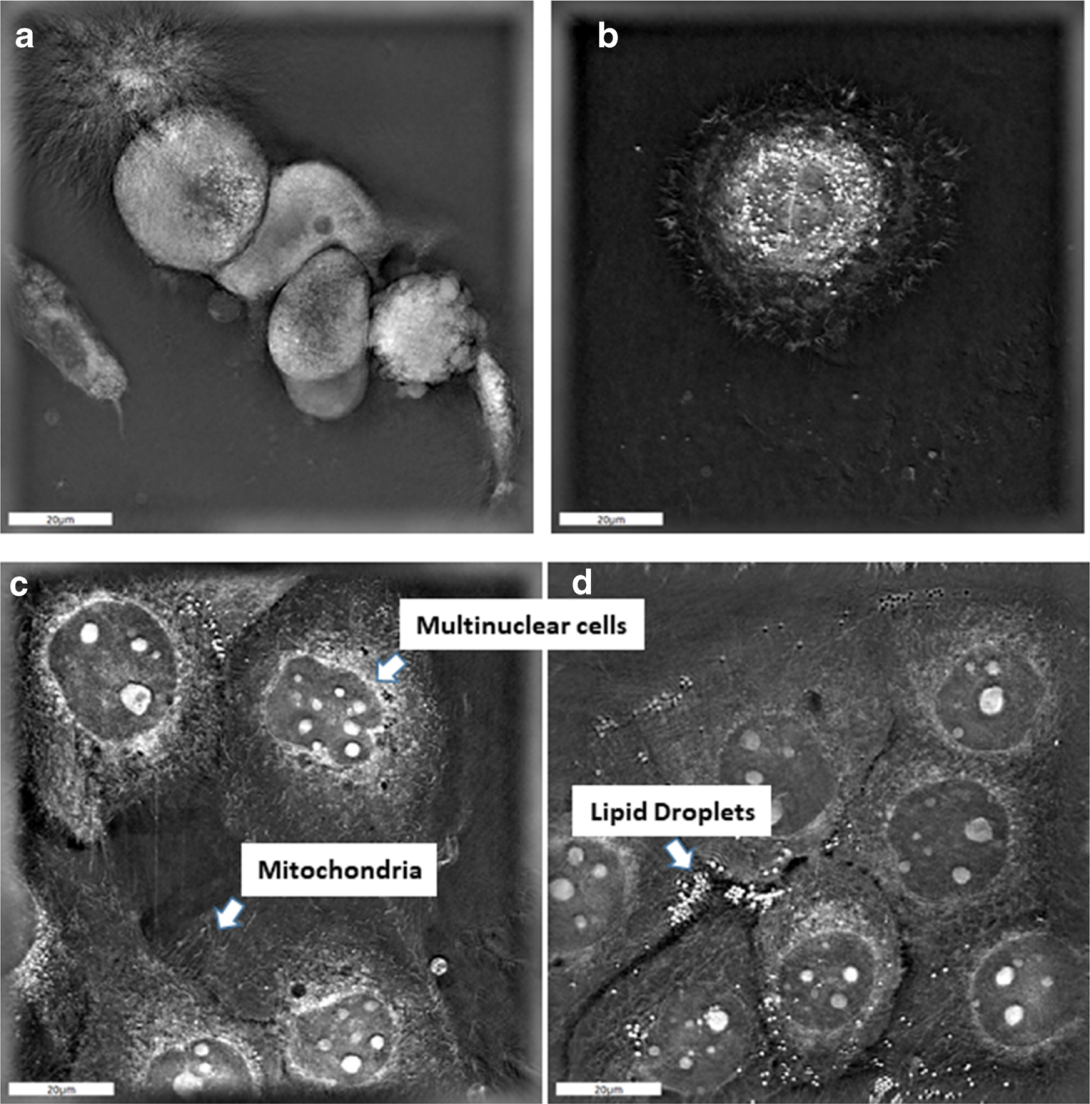
**EMC rescues cultured HNSCC cells from chemotherapy induced cell death.** As published by us before (4) EMC-conditioned media (EMC-CM) doubled the IC_50_ value of cisplatin in SCC-25 cells. If SCC-25 cells were treated with cisplatin at IC_50_ in albumin containing medium for 72 h the cells died and released their adhesion from culture dish (**A**, **B**), whereas, the SCC-25 treated with cisplatin at IC_50_ in EMC CM were alive, metabolically active and large. Furthermore the initiation of multinuclear morphology was observed with several mitochondria and lipid droplets (**C**, **D**). **A, B, C, D**: holotomographic microscopy images of SCC-25 cells using the NanoLIVE system. Scale bars 20 μm

As published by us before, treatment with EMC-CM increased the IC_50_ of Cisplatin in SCC-25 and Detroit 562 cells [4]. The IC_50_ of Cisplatin of SCC-25 cells increased from 6.2 μM to 13.1 μM (*p* < 0.001) in MTT assays after treatment with EMC-CM. The IC_50_ of Cisplatin of Detroit 562 cells was increased following treatment with EMC-CM from 13.1 μM to 26.8 μM (*p* < 0.01) [4]. These results were also confirmed by clonogenic assays, which are considered the gold standard for the assessment of chemotherapy or radiotherapy resistance in vitro [32]. Colony forming ability after treatment with 5 or 10 μM Cisplatin was significantly higher in HNSCC cells treated with EMC-CM than in controls (*p* < 0.05) [4]. These effects of EMC-CM on cisplatin resistance were visualized with holotomographic microscopy images of IC_50_ Cisplatin-treated SCC-25 cells using the NanoLIVE system. If SCC-25 cells were treated with cisplatin in albumin containing medium for 72 h the cells died and released their adhesion from the cell culture dish. In contrast, SCC-25 cells treated with the IC_50_ of cisplatin and EMC-CM, were still alive and metabolically active. Furthermore, the induction of a large, multinuclear morphology was observed with several mitochondria and lipid droplets (Fig. 5).

### EMC Effects Are Mediated by IL-6-STAT3 Signalling

To further elucidate the mechanistic backgrounds of EMC-CM the IL-6-STAT3 signalling cascade was hypothesized to be involved in the functions of EMC-CM, and it was investigated in more detail with western blot assays.

SCC-25 cells were either treated with EMC-CM or with media conditioned from a mono-culture of SCC-25 cells or HGF cells. SCC-25 cells constitutively synthesized STAT3 and Snail so these signalling factors could be found in all treatment arms. In contrast, phosphorylation of STAT-3 on Tyrosine 705 was only induced by EMC-CM treatment, which also achieved a significant induction of Slug protein synthesis.

On a second experimental setting the role of IL-6 in the observed effects from EMC-CM was analysed with western blots. The effect of EMC-CM on STAT3 phosphorylation was compared with the effect of medium conditioned from mono-cultures of SCC-25 or HGF and with albumin medium containing 50 ng/ml IL-6. STAT3 phosphorylation was only achieved by EMC-CM or by IL-6.

## Discussion

EMT in tumor progression is nowadays considered as a reversible process, which is reversed by its contrary process, mesenchymal to epithelial transition (MET). This phenotypic plasticity allows tumor cells to adapt to the different requirements of cancer progression [12]. EMT is closely linked to the induction of cancer stemness in metastatic disease allowing CSC to migrate to different organs. However MET, which increases proliferative capabilities of cancer cells in return is necessary, after CSC have reached their metastatic niche, for metastatic colonization of the host organ [6, 12, 33]. This dynamic model of EMT and MET is further supported by the finding of hybrid epithelial-mesenchymal cells which are favourable for the establishment of metastasis. These hybrid phenotypes could be found in circulating tumor cells from patients with non-small cell lung cancer [34], prostate cancer [35] and breast cancer [36–38]. This phenotypic plasticity was described as stemness related feature, as these hybrids cells were demonstrated to express high levels of CSC markers, too [35, 39].

**Fig. 6:**
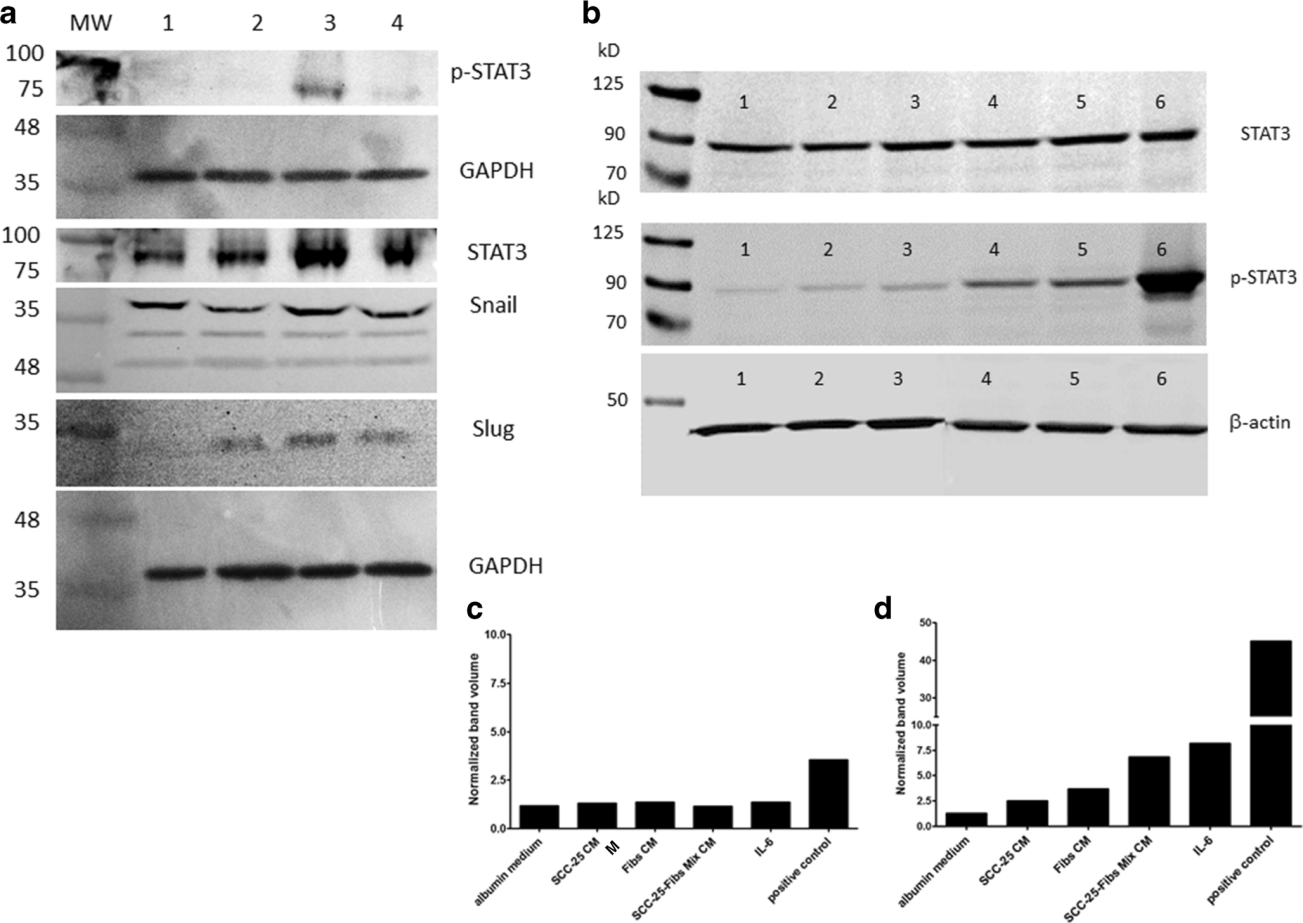
**EMC-CM transmits induction of EMT through IL-6 - STAT3 signalling.** SCC-25 cells were treated with conditioned media of single culture of SCC-25 cells (**A**, 1), single culture of HGF fibroblasts (**A**, 2), mixed EMC culture (**A**, 3), or were indirect co-cultured with HGF fibroblasts (**A**, 4). **A**) Western blot analysis of treated SCC-25 for STAT3, phospho-STAT3, Snail and Slug. GAPDH was used as loading control. The SCC-25 cells constitutively synthesized STAT3 and Snail. STAT-3 was only phosphorylated by mixed EMC-CM treatment, which also achieved a significant induction of Slug protein synthesis. **B**) Western blot analysis of treated SCC-25 cells for STAT3, phospho-STAT3. β-actin was used as loading control. 1: cells cultured in albumin-containing medium, 2: cells treated with CM of single culture of SCC-25 cells, 3: cells treated with CM of single culture of HGF fibroblasts, 4: cells treated with CM of mixed EMC culture, 5: cells treated with 50 ng/ml IL-6, 6: commercial available positive control of induced STAT3 phosphorylation. STAT3 phosphorylation was achieved by CM of mixed EMC culture or by IL-6. **C-D**) Normalized optical density quantification of western blots presented in **B, C:** STAT3**, D:** phospho-STAT3 (*n* = 3)

In this study a cell culture model of EMT was established in the mixed culture of epithelial tumor cells and fibroblasts as epithelial – mesenchymal crosstalk (EMC). The treatment with EMC-CM induced a hybrid phenotype in naïve SCC-25 cells and also the protein synthesis of EMT-related transcription factors as Snail or Slug (Fig. 6). Furthermore, this hybrid phenotype could be found in the invasive front of tissue biopsies from patients with head and neck cancer (Fig. 3). These findings might be clinical relevant as we were able to demonstrate before that EMC-CM led to chemotherapy and radiotherapy resistance in HNSCC cells, indicating that this hybrid epithelial-mesenchymal phenotype might be involved in therapy resistance, which is also described as a key feature of CSC [4, 5, 40].

Visualisation of these EMC-induced hybrid cells with holotomographic microscopy after exposure to 6.2 μM cisplatin showed that these cells survived cisplatin treatment and a large, multinuclear morphology was induced with several mitochondria and lipid droplets. Native SCC-25 in contrast died and released their adhesion from the cell culture dish (Fig. 5). Similarly to previous studies these EMC-induced hybrid cancer cells showed a higher proliferation and consequently tumor-initiating ability (Fig. 4) [41, 42].

As a possible mediator of these effects we suggested IL-6, as it is the most abundant cytokine in EMC-CM. IL-6 has been shown to regulate cancer cell stemness in several studies [43, 44]. IL-6 binding to its receptor induces activation of Jak/Stat3 signaling, involved in epithelial-to-mesenchymal transition (EMT) [44, 45].

Furthermore, the maintenance of a dynamic equilibrium between CSCs and non-CSC has been suggested to be balanced by the amount of IL-6 secreted by CSCs meaning that via IL-6 signalling either CSC differentiation or self-renewal of CSC can be promoted [46]. In our experimental setting only EMC-CM induced IL-6 dependent STAT3 phosphorylation of SCC-25 cells suggesting IL-6 as mediator balancing this EMC-related hybrid phenotype (Fig. 6).

## Conclusion

EMC conditioned medium led to the induction of a hybrid epithelial-mesenchymal cancer cell phenotype, characterised by co-expression of vimentin and cytokeratins. This phenotype is associated with increased self-renewal, therapy resistance and migration and can also be found in the invasive front of tissue biopsies from head and neck cancer patients. These observed effects might be mediated by IL-6-STAT3 signalling.
